# Supplementary material for: Nkx2.5 marks angioblasts that contribute to hemogenic endothelium of the endocardium and dorsal aorta
Source: eLife. 2017 Mar 8;6:e20994. doi: 10.7554/eLife.20994 (PMC5400512; doi:10.7554/eLife.20994)
Supplement: Figure 4—source data 1. — Analysis is based on six embryos from two independent experiments. The first column represents the tissues checked for Hb-en+ cells. The second column represents the percentage of embryos in which Hb-en+ cells were detected. The third column represents the percentage of double positive Hb-en+/CD45+ cells out of the total CD45+ cells. DOI: http://dx.doi.org/10.7554/eLife.20994.012 [file elife-20994-fig4-data1.docx]

| Embryonic tissue | Detection of Hb-en^+^ cells | Hb-en^+^/CD45^+^ |
| --- | --- | --- |
| Dorsal aorta | 4/6, ~67% | ~48% |
| Endocardium | 6/6, 100% | ~28% |
| Cardinal veins | No | - |
| Extraembryonic/LPM | 6/6, 100% | - |

**Figure 4 – source data 1.** The distribution of Hb-en^+^ cells in the chick embryo. Analysis is based on six embryos from two independent experiments. The first column represents the tissues checked for Hb-en^+^ cells. The second column represents the percentage of embryos in which Hb-en^+^ cells were detected. The third column represents the percentage of double positive Hb-en^+^/CD45^+^ cells out of the total CD45^+^ cells.
